# An Account of the Amputations Performed in the Pennsylvania Hospital, from March, 1836, to March, 1839

**Published:** 1839-03-16

**Authors:** H. H. Smith

**Affiliations:** Resident Surgeon


### CLINICAL REPORTS.

### PENNSYLVANIA HOSPITAL.

*An account of the Amputations performed in the Pennsylvania Hospital, from March*, 1836, *to March*, 1839.

[Reported by H. H. Smith, M. D., Resident Surgeon.]

The frequent occurrence of cases requiring amputation in all large hospitals, has lately drawn the attention of the profession to the results; the opinions of those most immediately concerned in the performance of them, having tended to the support of the idea that a large proportion terminate fatally. This opinion has not unfrequently, it is feared, led to attempts at saving limbs, which offered very little prospect of success, rather than submit the patient to a pain so severe as that consequent on the performance of the operation with so slight a prospect of a happy termination, judging from the tabular statements. How far this method of testing their result may be correct, is left to others to determine. But when we consider the difference of circumstances under which the operation may be necessary, reason demands that some reference should be made to the cause, habits of life, climate, season and manner of dressing, in order to unable us to form an opinion as to the prospect of a successful termination in even the best case. With this view the following account of the amputations performed by the surgeons of the hospital during three years is offered, not that the period is considered as one of sufficient length for a general average, but because a statement of each case has been preserved from a time previous to the operation up to that of the cure, the summary of which is here offered according to the year in which they were performed.

*Amputations during the year, from March*, 1836, *to March*, 1837.

Case I.—Joseph S——, labourer, aged 26 years; admitted March 11th, 1836, for a compound fracture of the leg, caused by a wall falling on him. Leg amputated below the knee on same day. Ulceration of flap, from the pressure on the sharp extremity of the tibia, delayed cure. Discharged cured, June 25th, one hundred and six days after the amputation.

Case II.—Patrick G——, labourer, æt. 38 years; admitted March 2d, for frost-bite of both feet—general health good—was in the house some time, before the operation. One foot was disarticulated at metatarsal bones and healed first. The other was sawed off near the same point. Cured July 6th, one hundred and twenty-six days after admission.

Case III.—Henry T——, sailor, æt. 22 years; admitted March 5th, with frosted feet, partial amputation of toes of one foot by disarticulation, after sloughing ceased—discharged cured, September 8th, near six months after admission.

Case IV.—Francis McI——, labourer, æt. 21 years; admitted June 12th, 1836, for a compounl fracture of both bones of leg three inches above joint—two inches of the tibia were removed at entrance, and this leg got well. The other foot was much mangled, a locomotive and cars having passed over him, and was amputated below the knee immediately. He was an habitual drunkard—had several attacks of mania potu, and also erysipelas, but was discharged cured, January 25th, 1837, near seven months after the accident. He has been seen within a few days (March 5th, 1839;) small pieces of bone keep an ulcer yet open on leg.

Case V.—John B——, æt. 6 years, was admitted July 23d, having been run over on a rail road. His thigh had been amputated close to seat of injury, by a person in the neighborhood, but was not dressed when he entered the house. It was therefore dressed, but sloughed from contusion of surrounding parts. He was discharged cured, December 7th, near five months after the amputation.

Case VI.—Joseph M——, farmer, æt. 46 years, was admitted with a severely lacerated foot, from injury by a locomotive engine. Foot amputated by disarticulation at the os calcis eight hours after admission. Habits fair—but had had an attack of paralysis some time previous to accident. He never re-acted fully, and died seven days after admission.

Case VII.—John K——, sailor, æt. 22 years, was admitted September 2d, for caries of the foot and ulcers, scrofulous habit, erysipelas attacked whole limb—had slough on his sacrum, and was much reduced previous to amputation, which was not done till some time after admission. Discharged cured, April 15th, 1837, over seven months after his entrance.

Case VIII.—William C——, tailor, æt. 47 years, was admitted December 9th, with a compound fracture of the knee-joint, from an injury by a locomotive. Habits bad—drunk at time of accident—thigh amputated immediately—died within 24 hours afterwards.

Case IX.—Robert B——, tailor, æt. 50 years; fell in between two iron rollers in a cotton factory, and was drawn up till his thigh was torn off. When admitted, February 27th, 1837, the vessels and nerves were exposed and ragged—

the stump was trimmed and dressed, and he died five hours after admission.

For the above cases I am indebted to the notes of my friend Dr. J. M. Wallace, at the time residing in the house. From these it will be seen that but three cases out of nine died during this year—in none of which was the prospect of cure at all favourable.

*Amputations from March*, 1837, *to March*, 1838.

Case I.—Thomas D——, æt. 41 years, was admitted February 1st, with a large leg ulcer, which had lasted near ten years; sloughing ensued which was followed by caries of the knee-joint. The thigh therefore was amputated about six weeks after his entrance, to save his life, but without success, death occurring three days after the operation.

Case II.—John D——, labourer, æt. 25 years; admitted August 19th, for compound fracture of the radius, caused by a tight bandage, which rendered the previous simple fracture compound—sloughing followed, and the arm was amputated above the elbow September 11th. Discharged cured, November 25th, near eleven weeks after admission.

Case III.—William P——, labourer, æt. 45 years; admitted September 9th, for caries of the wrist-joint. Amputation of the forearm was performed September 26th, and he was discharged cured, November 27th, four weeks after the operation.

Case IV.—William H——, weaver, æt. 29 years, was admitted October 10th, for a gun shot wound of the hand, the whole load having passed through the palm—sloughing ensued and opened the wrist-joint, so that amputation below the elbow was performed October 28th. Discharged cured, November 30th, thirty-three days after the operation. The stump ulcerated after his discharge, but he recovered.

Case V.—Leah L——, coloured, æt. 24 years; married, was admitted October 24th, for a fungus hematodes tumour on the thigh. General health fair. Thigh amputated high up November 15th, afterwards opened on account of secondary hæmorrhage. Discharged cured, January 10th, 1838—eight weeks after the operation. This patient died September 17th, 1838, after residing in the city, and a post-mortem examination showed the stump to be perfectly healthy, the end of the bone nicely rounded off, but the lungs were filled with the encephaloid matter similar to that originally in the tumour.

Case VI.—Absalom L——, coloured, æt. 29 years, was admitted November 17th, for a compound fracture and dislocation of the arm. The hand was caught in the machinery of a steam biscuit-bakery and drawn in up to the shoulder. The few shreds holding the arm together at its middle, were divided, and the vessels tied immediately. Four hours afterwards it was amputated at the shoulder joint, and he died three days after this, having never reacted fully.

Case VII.—David L——, coloured æt. 24 years, was admitted October 14th, for caries of the tarsal bones, consequent on a sprain two years previously. The leg was amputated below the knee, January 24th, 1838, and he was discharged cured, February 21st—four weeks after the operation.

Of the seven cases amputated this year, but two died after the operation—the cause of the amputation being such as to explain the termination of it.

*Amputations from March*, 1838, *to March*, 1839.

Case I.—Peter C——, shoemaker, æt. 22 years, was admitted December 20th, 1837, for caries of the knee, consequent on a sprain and the use of his hammer on it in pursuing his trade. The thigh was amputated February 28th, 1838, and on March 28th he was discharged cured, one month after the operation.

Case II.—Hugh F——, labourer, æt. 31 years, was admitted April 10th, for a compound fracture of the elbow-joint, caused by blasting—the rod being driven through the joint. The arm was amputated immediately, although much inflamed from a burn from powder, but united beautifully in three weeks after the operation. He was treated then for ophthalmia, consequent on same injury, and not discharged until July 16th, the stump continuing well.

Case III.—Samuel N——, sailmaker, æt. 16 years, was admitted March 29th, for a compound fracture of the elbow from a gun shot wound. The arm was amputated April 25th, neuralgia of the stump ensued, and exfoliation of bone for two inches, and he was discharged July 21st, eighty-seven days after the operation.

Case IV.—Samuel J——, labourer, æt. 39 years, was admitted August 14th, for a compound fracture of the hand and arm, caused by its being drawn in between two rollers; sloughing followed, and September 12th amputation was performed above the elbow. Discharged cured, October 31st, forty-nine days after the amputation.

Case V.—Isaac W——, labourer æt. 40 years, was admitted Sept. 14th, for conpound oblique fracture of both bones of the leg;—drunkard, and intoxicated at the time of falling; mania potu followed, in which it sloughed, and on October 6th it was amputated below the knee; healed well, and on Dec. 10, sixty-five days after the operation, he was discharged cured.

Case VI.—Peter McG——, labourer æt. 66 years, was admitted Oct. 22d, for a compound fracture and dislocation of the ankle, caused by a rail road car passing over him. The integuments were much torn, and it was amputated near the knee, a few hours after his admission. Discharged cured Dec. 10th, forty nine days after the operation.

Case VII.—Samuel C——, labourer, æt. 23 years, was admitted Oct. 10th, for a compound fracture of the knee, caused by a rail road car. Violent inflammation followed, and it was amputated at the thigh Nov. 7th, and discharged cured Dec. 13th, 6 weeks after the operation. An examination of the leg showed an oblique fracture of the lower part of the thigh, and two oblique fractures in the condyles.

Case VIII.—James R——, æt. 12 years, was admitted Dec. 4th, with a laceration and compound fracture of the arm and hand, from its being caught in machinery; amputated 4 hours after entrance—had three distinct attacks of erysipelas, but was discharged cured Feb. 16th, with the prospect of partial exfoliation of the bone.

Case IX.—Robert H——, weaver, æt. 30 years, was admitted Nov. 30, with an old unreduced dislocation of the hip, which also anchylosed as well as the knee, the latter being bent was in his way when at work, which induced him to come from Reading to have it removed. This was decided on and done Dec. 6th; the stump had nearly healed, when it was attacked by erysipelas which caused sloughing of the integuments, and left the bone projecting near 3 inches.

Case X.—Joseph C——, sailor, æt. 43 years, was admitted Oct. 10th, for fracture of three fingers; he was attacked with the mania potu—then with erysipelas, the sloughing from which opened the metacarpal joint of the first finger; this was amputated Dec. 12th, and he was cured Feb. 20th, 1839, having had two subsequent attacks of erysipelas.

Case XI.—Mason M——, æt. 15 years, was admitted Jan. 16th, for a compound dislocation and fracture of the ankle, caused by machinery; amputation was performed immediately, two attacks of erysipelas followed, but it is now, March 5th, cicatrizing.

Case XII.—Anthony S——, printer, æt. 45 years, was admitted Jan. 4th, 1839, for a laceration and contusion of the hand, caused by machinery; it was necessary to tie both the radial and ulnar arteries; erysipelas followed, and sloughing, so as to render it necessary to amputate the forearm Feb. 13th, 1839. March 5th, it has nearly cicatrized.

Case XIII.—Robert H——, re-amputation of case No. 9, by the *flap operation*, Feb. 20, 1839, has nearly cicatrized, March 5th.

Case XIV.—John E——, æt. 50 years, was admitted Feb. 4th, for an incised wound of the wrist; afterwards attacked by erysipelas, rendering amputation of the forearm necessary; Feb. 23, the ligatures are all away, and it is, March 5th, healing rapidly.

Of the fourteen cases operated on this year, none have terminated fatally, although some have been thrice attacked by erysipelas. The whole number amputated during the three years of 1836-37-38 and 39, has been 30.

| | | | | |
|---|---|---|---|---|
| Of which there have been of the | | | Thigh | 9 |
| " | " | " | Leg | 7 |
| " | " | " | Foot | 4 |
| " | " | " | Arm | 5 |
| " | " | " | Forearm | 4 |
| " | " | " | Finger | 1 |
| Of this number there have been cured | | | | 25 |
| " | " | " | Died | 5 |

As a full account has been given of the causes of injury, state of the patients &c., it will be easy to form a correct judgment of the actual mortality to be ascribed to the operation; many of the latter cases have had several attacks of erysipelas, but none have terminated fatally. The few not entirely well, are so near it as to cause no doubt of the termination. When the varied kinds of accidents are considered, and the large proportion due to machinery and rail roads, which generally mangle the limb horribly, the success has certainly been great. What might be the result of the addition of a few years to this, I can not say, but at present the average of deaths is small.

